# Acupoints Stimulation for Anxiety and Depression in Cancer Patients: A Quantitative Synthesis of Randomized Controlled Trials

**DOI:** 10.1155/2016/5645632

**Published:** 2016-03-29

**Authors:** Tao Wang, Renli Deng, Jing-Yu Tan, Feng-Guang Guan

**Affiliations:** ^1^The Fifth Affiliated (Zhuhai) Hospital of Zunyi Medical University, No. 1439, Zhufeng Road, Zhuhai, Guangdong 519100, China; ^2^School of Nursing, Fujian University of Traditional Chinese Medicine, No. 1, Qiuyang Road, Fuzhou, Fujian 350122, China; ^3^The Second Affiliated People's Hospital, Fujian University of Traditional Chinese Medicine, No. 282, Wusi Road, Fuzhou, Fujian 350003, China

## Abstract

This study aims at concluding the current evidence on the therapeutic effects of acupoints stimulation for cancer patients with anxiety and depression. Randomized controlled trials using acupoints stimulation for relieving anxiety and/or depression in cancer patients were searched, and 11 studies were finally included, of which eight trials compared acupoints stimulation with standard methods of treatment/care, and acupoints stimulation showed significantly better effects in improving depression than using standard methods of treatment/care. Four studies compared true acupoints stimulation with sham methods, and no significant differences can be found between groups for either depression or anxiety, although the pooled effects still favored true intervention. For the five studies that evaluated sleep quality, the results were conflicting, with three supporting the superiority of acupoints stimulation in improving sleep quality and two demonstrating no differences across groups. Acupoints stimulation seems to be an effective approach in relieving depression and anxiety in cancer patients, and placebo effects may partially contribute to the benefits. However, the evidence is not conclusive due to the limited number of included studies and the clinical heterogeneity identified among trials. More rigorous designed randomized, sham-controlled studies are necessary in future research.

## 1. Introduction

Cancer is one of the major health problems in the world. The incidence of cancer and the related death have significantly increased during the past decades. According to the global corresponding data reported by the International Agency for Research on Cancer (IARC), 14.1 million cancer patients were identified around the world and 8.2 million patients died in 2012 [1]. Cancer-related death has gradually surpassed the relative figure of all types of heart disease and stroke and has become the leading cause of death at present [2].

With the advance of medical technologies, cancer treatments have changed reasonably, and comprehensive treatment strategies including optimized pharmacological intervention [3] and palliative care can ease most physical symptoms effectively and let cancer survivors live a relatively symptom-free life [4, 5]. Although the survival rate has been rising over the past decade, the negative cancer experiences and the side effects associated with cancer treatments (e.g., pain, fatigue, nausea, and vomiting) can result in a wide range of health problems, which contain not only physical issues but also psychological and emotional distresses including depression, anxiety, and posttraumatic stress disorder (PTSD) [5–9]. The incidence of depression in cancer survivors is three to four times as much as that in general population [10], ranging from 10% to 50% [11, 12]. The percentages of anxiety and PTSD following cancer were reported to be 6% to 23% [13] and 0% to 32% [14], respectively. The negative emotional feelings can contribute to significantly undesirable impacts on cancer patients, leading to sleep disturbance, loss of appetite, deterioration of quality of life, and so forth [15]. In turn, the psychological and emotional disorders can also increase the risk of recurrence and mortality of cancer as psychological distresses could impair patients' immune response [16].

Healthcare professionals have recognized that cancer treatment should aim at not only expanding patients' life span but also improving their psychosocial well-being [17]. In recent years, healthcare professionals and patients are increasingly interested in using complementary therapies to relieve cancer survivors' emotional problems such as anxiety and depression and make them as alternatives to the mainstream medicine [18]. Acupoints stimulation as one kind of complementary therapies originates from ancient China, and it is based on the theory of main and collateral channels. For the modalities of acupoints stimulation, both acupuncture and acupressure are used in practice. The World Health Organization (WHO) has viewed acupoints stimulation as a beneficial intervention to deal with a wide range of health problems [19], and its therapeutic effects for psychological distresses, particularly with regard to anxiety and depression, have been explored in noncancer populations, and corresponding systematic reviews and clinical trials have been conducted to identify its positive effects [20–23]. Cancer patients as the special population suffer more from anxiety and depression [10], and acupoints stimulation can be a promising therapy, but the evidence remains unknown.

Preclinical research has indicated that both the hypothalamic-pituitary-gonadal axis (HPA) and the sympathetic nervous system (SNS) can be significantly affected by acupoints stimulation, which presents a possible explanation that acupoints stimulation may have a positive impact on reducing psychological distresses from the biological perspectives [24, 25]. A large number of clinical trials have also been conducted to explore whether acupoints stimulation is effective in controlling anxiety and depression in cancer patients but their findings were conflicting [26–36]. Some demonstrated that acupoints stimulation can effectively relieve anxiety and/or depression while some did not. The clinical heterogeneity in patient characteristics, intervention protocols, and outcome variables and the various degrees of methodological quality among those trials may partially contribute to the contradictory study results. However, to our knowledge, currently there is no systematic summary in regard to the methodological quality assessment of the trials on acupoints stimulation for anxiety and depression in cancer patients, and the overall evidence on its clinical efficacy remains uncertain. Therefore, the objectives of this systematic review are to explore the current evidence on acupoints stimulation for anxiety and depression in cancer patients and to offer recommendations for future research and practice.

## 2. Methods

### 2.1. Search Strategies

We retrieved relevant references by searching electronic databases. Ten databases were searched (up to November 20, 2014) which include PubMed, Cochrane Central Register of Controlled Trials (CENTRAL), Cumulative Index to Nursing and Allied Health Literature (CINAHL), Allied and Complementary Medicine (AMED), PsycINFO, ISI Web of Science, Science Direct, WanFang Data, China National Knowledge Infrastructure (CNKI), and Chinese Scientific Journal Database (VIP). When conducting the electronic search, we did not set restrictions for the publications in terms of types of language and types of study. Mesh terms and key words used in the search strategies were “acupuncture”, “acupressure”, “acupuncture, ear”, “electroacupuncture”, “neoplasms”, “anxiety”, “depression”, “mood disorders”, and so forth; we also searched the references of the final included articles and other Chinese core journals on Traditional Chinese Medicine (TCM) to see whether we can locate any publications for possible inclusion. The literature search was conducted independently by two reviewers. In Table 4, we list two search strategies for this systematic review.

### 2.2. Study Selection and Data Extraction

After completing literature search, the same two reviewers independently screened the title and abstract of all searched articles, and the final eligible articles were obtained and read in full text. Inclusion criteria for eligible studies were (1) randomized controlled trials comparing acupoints stimulation (including manual acupuncture, electroacupuncture, or acupressure) to one or more of the following situations: sham acupoints stimulation, standard treatment/care (usual care, standard medication, and attention control group were all defined as standard treatment/care in this systematic review), or wait-list control; (2) participants who should be cancer patients with anxiety and/or depression regardless of being children or adults; and (3) trials published in English or Chinese.

The primary outcome measures were anxiety and depression as measured by Hospital Anxiety and Depression Scale (HADS), Center for Epidemiologic Studies Depression Scale (CESD), Hamilton Depression Scale (HAMD), Beck Depression Inventory-Primary Care (BDI-PC), and so forth, while the secondary outcome was the status of sleep quality as measured by Pittsburgh Sleep Quality Index (PSQI), sleep log, and so forth. Characteristics of the included studies were extracted through a data extraction form which was piloted prior to the commencement of the study.

### 2.3. Methodological Quality Assessment for the Included Studies

We evaluated the methodological quality of each included trial with the 2015 risk of bias criteria provided by the Cochrane Back and Neck (CBN) Group [37], which include 13 specific domains: random sequence generation, allocation concealment, blinding of participants, blinding of caregiver, blinding of outcome assessor, description and acceptance of dropout rate, intention-to-treat analysis, selective outcome reporting, baseline similarity, cointerventions, acceptable compliance, timing of outcome assessment, and other sources of bias (e.g., validity of the outcome assessments and the report of the conflict of interest). The risk of bias criteria used in this study are adapted from the Cochrane Handbook of Reviews of Interventions and are appropriate for studies using nonpharmacological intervention [37]. Two reviewers independently assessed the risk of bias of the included RCTs. Each of the 13 risk of bias items was rated as “low risk of bias,” “unclear risk of bias,” or “high risk of bias.” Methodological quality assessment was further checked by a third review author and any disagreements on the risk of bias judgment were handled by discussion.

### 2.4. Data Analysis

We used the Review Manager 5.3 (Cochrane Collaboration) to conduct the data synthesis. Regarding the continuous outcome variables, we calculated the standardized mean difference (SMD) or the weighted mean difference (WMD) with 95% confidence intervals (CI). Fixed effect model was considered when the trials had a satisfactory statistical homogeneity which was evaluated by examining *I* ^2^ (*I* ^2^ < 50%). Otherwise, a random effect model was applied. Both overall assessment and subgroup estimation were performed. The overall assessment on the effects of acupoints stimulation for mood problems and sleep quality was performed first regardless of the acupoints stimulation types. Subgroup analysis based on different acupoints stimulation modalities (manual acupuncture, electroacupuncture, and acupressure) was carried out afterwards. If the data synthesis could not be conducted owing to the absence of available data, different types of data, or a significant heterogeneity in the outcome effect, descriptive analysis was considered instead.

## 3. Results

### 3.1. Characteristics of the Included Studies

**Figure 1:**
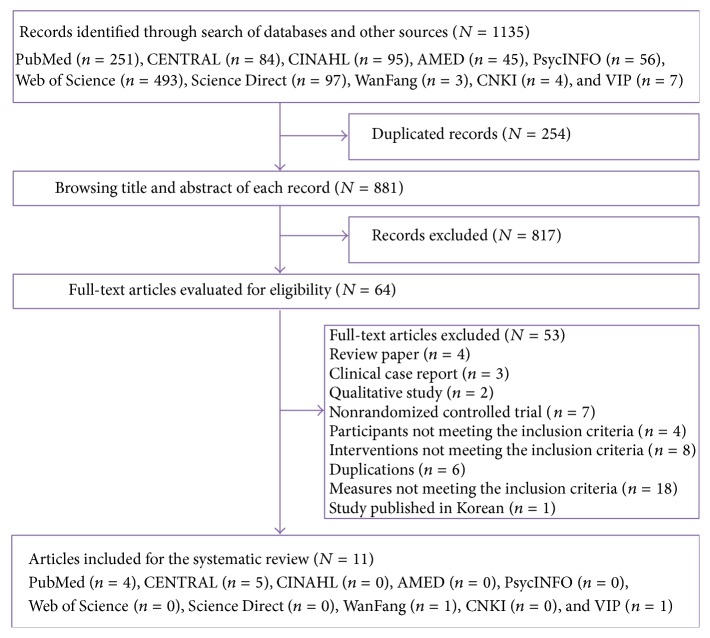
Flow chart of study selection.

A total of 1135 potential records were yielded by searching the electronic databases, and 11 trials [26–36] were finally retrieved for systematic review. The flow chart (Figure 1) presents the selection process of the qualified studies. The 11 included studies, with nine English and two Chinese articles, were all journal papers published between 2006 and 2014. The study sample ranged from 30 to 302 and the average sample size was 98. A total of 1073 participants (number of randomized participants) with various types of malignancies were involved. Six studies focused on breast cancer [26, 29, 31–34], one study was on lung cancer [30], one study was on gynecological cancer [36], and three studies [27, 28, 35] involved more than two types of malignancies. For the study design, eight [26–28, 31–33, 35, 36] were two-arm design and the other three [29, 30, 34] studies included three study arms.

Anxiety and/or depression were assessed in 11 studies. Seven of them assessed anxiety, out of which six studies [26, 27, 29, 30, 33, 34] used Hospital Anxiety and Depression Scale (HADS-A) which is commonly used in clinical research related to emotional problems in cancer [38] and the other one [32] adopted Psychological and General Well-Being Index (PGWB) which is a validated quality of life (QoL) instrument and “anxiety” is included as one of the subscales. Eleven studies assessed depression, out of which one study [26] employed CESD, one [36] applied HAMD only, two [28, 35] used both HAMD and Self-Rating Depression Scale (SDS) (only data from HAMD was used for data synthesis in order to ensure the homogeneity of outcome measures among trials), five [27, 29, 30, 33, 34] employed HADS subscale for depression (HADS-D) only, and two studies adopted BDI-PC [31] and Psychological and General Well-Being Index (PGWB) [32], respectively. Sleep quality was assessed in five studies [26, 28–30, 32] and the PSQI was employed in four trials [26, 28–30], while in the other one study [32], sleep quality was measured by recording the times of wake-up and the hours of sleep on log book. Characteristics of the included studies are presented in Table 1.

### 3.2. Description of the Intervention Protocol

Intervention protocols used in the included studies are also summarized in Table 1. Three acupoints stimulation modalities were employed, of which seven [26–28, 31, 33, 34, 36] adopted manual acupuncture and three [29, 32, 35] used electroacupuncture while only one trial [30] chose acupressure. Of the seven studies that adopted manual acupuncture, two [26, 27] assessed its therapeutic effects by comparing true acupuncture with sham acupuncture (selected sham acupoints were located away from the true acupoints), four studies [28, 31, 33, 36] compared true acupuncture with standard treatment/care (two [28, 31] adopted standard medication, specifically antidepressant, as the standard treatment and the remaining two [33, 36] used attention control or usual care), and one trial [34] with three-arm design investigated the effects of acupuncture by comparing the true acupuncture which was conducted by acupuncture practitioners with two types of controls: the self-acupuncture control conducted by the participants and the standard treatment/care control without acupuncture. Of the three studies that used electroacupuncture, one compared true electroacupuncture with either sham electrostimulation or wait-list control [29] and the other two studies [32, 35] compared true electroacupuncture with standard treatment/care (standard medication in one study [32] and usual care in the other [35]). The only one study [30] employing acupressure was a three-arm design, with one group using acupressure plus oil and the other two adopting true acupressure and sham intervention, respectively. For this study, we only abstracted the data from the true and sham acupressure groups for analysis. Ten studies described the protocols of acupoints stimulation, which included the selection and identification of targeted acupoints, types of stimulation, and duration and frequency of treatment. Regarding the selected acupoints,* Sanyinjiao* (SP6),* Zusanli* (ST36), and* Hegu* (LI4), which can be identified in nearly half of the included studies, were the most frequently adopted acupoints for depression and/or anxiety.

### 3.3. Methodological Quality and Risk of Bias of the Included Trials

Table 2 shows the results of the risk of bias assessment for the included studies. Methodological quality of the trials was generally acceptable. All of the analyzed trials mentioned the method of randomization, and details of producing the random sequence were reported in nine studies by applying random number table, using computer software, or tossing coin. Treatment allocation was reported to be concealed in only five studies. Blinding of participants, care provider, or outcome assessor was performed in four studies. All studies reported the dropout of study subjects and in ten of them the dropout rates were deemed as acceptable (less than 20%) [37], whereas, in the other one study, the dropout rate exceeded 30% in the long-term observation (high risk of bias). In eight trials, all subjects with randomization were included for data analysis based on missing data handling approach such as intention-to-treat analysis. Only one study [31] failed to specify whether it had selectively reported study outcomes or not. All studies were similar at baseline regarding the major demographic variables, and the timing of the outcome assessment was also reported to be similar in all the included trials.

### 3.4. Therapeutic Effects of Acupoints Stimulation

Results of the data synthesis for the systematic review are summarized in Table 3.

#### 3.4.1. Overall Assessment of Acupoints Stimulation for Anxiety and Depression

*(i) Acupoints Stimulation versus Standard Methods of Treatment/Care*. Eight studies [28, 29, 31–36] compared the effects of acupoints stimulation with standard methods of treatment/care. All of the eight trials evaluated depression, four [29, 32–34] measured anxiety, and three [28, 29, 32] measured sleep quality. Meta-analysis indicated that acupoints stimulation can significantly relieve depression in cancer patients [random effect model, SMD = −1.08, 95% CI = −1.97 to −0.19, and *P* = 0.02]. However, statistical heterogeneity (*P* < 0.0001, *I* ^2^ = 94%) was identified in the pooled effects, and removal of any suspicious trials did not change the heterogeneity significantly. For the eight studies that evaluated depression, two trials [28, 31] compared acupuncture with standard medication (antidepressant), and data synthesis was not conducted due to different kinds of outcome measures. According to the descriptive analysis of each single trial, acupoints stimulation (acupuncture) demonstrated a similar [31] or even better effect [28] in controlling depression than using antidepressant.

Data synthesis cannot be conducted for anxiety due to the insufficient number of studies and the inconsistent types of data. Descriptive analysis was adopted instead. Two trials [29, 33] showed significant improvements in anxiety. In the study conducted by Mao et al. [29], acupoints stimulation (electroacupuncture) was found to be effective in relieving anxiety in cancer survivors (*P* = 0.044) after ten treatments over eight weeks. There are two articles [33, 34] reporting two different study phases from one large trial. In one paper published in 2012 [33], a 6-week acupuncture treatment led by the acupuncturists was compared with enhanced usual care (attention control) which was implemented by providing patients with an information booklet to maintain the consistency of the control group, and the results favored acupuncture in reducing anxiety (*P* < 0.01). Sample from the acupuncture group was rerandomized in a further trial [34] to compare the effects of 4-week acupuncturist-delivered acupuncture with either 4-week self-acupuncture or no maintenance group (usual care), and no marked differences in anxiety scores were detected across groups.

For sleep quality, conflicting study results were reported where one study [28] favored acupoints stimulation (acupuncture) in improving the quality of sleep (*P* < 0.01), while another one [29] reported that acupoints stimulation (electroacupuncture) failed to contribute to significant improvement in PSQI score (*P* = 0.058) when comparing with the wait-list control.

*(ii) Acupoints Stimulation versus Sham Acupoints Stimulation*. Acupoints stimulation was compared with sham intervention in four trials [26, 27, 29, 30]. All of them involved anxiety and depression and three [26, 29, 30] assessed sleep quality. For anxiety and depression, only two studies were eligible for synthesis. Although the differences between groups did not reach a statistical significance, the pooled effects still favored true acupoints stimulation in relieving either anxiety [random effect model, MD = −0.65, 95% CI = −2.84 to 1.53, and *P* = 0.56] or depression [random effect model, MD = −0.27, 95% CI = −2.66 to 2.12, and *P* = 0.82]. For sleep quality, descriptive analysis from one trial [30] indicated that acupoints stimulation (acupressure) could improve sleep quality (*P* = 0.040).

#### 3.4.2. Subgroup Analysis on Manual Acupuncture

*(i) Manual Acupuncture versus Standard Methods of Treatment/Care*. Manual acupuncture on anxiety and/or depression was compared with standard methods of treatment/care in five studies [28, 31, 33, 34, 36], and manual acupuncture was found to be superior in depression relief [random effect model, MD = −2.55, 95% CI = −3.61 to −1.49, and *P* < 0.00001]. However, significantly statistical heterogeneity (*P* = 0.002, *I* ^2^ = 80%) was identified in the pooled effect. This might be caused by Feng et al.'s study [28] as the *I* ^2^ decreased to 0% (*P* = 0.89) after removing the mentioned trial, and the pooled effect was relatively stable, which still favored manual acupuncture [random effect model, MD = −2.10, 95% CI = −2.56 to −1.64, and *P* < 0.00001]. Descriptive analysis also supported the superiority of acupuncture in managing anxiety (*P* < 0.01) [33] and improving sleep quality (*P* < 0.01) [28] in cancer patients.

*(ii) Acupuncture versus Sham Acupuncture*. Sham acupuncture was adopted in two studies [26, 27]. Due to different types of data, we used descriptive analysis here. One study [26] indicated that there were no significant differences for between-groups comparison in depression (*P* = 0.442), anxiety (*P* = 0.526), and sleep quality (*P* = 0.557). While for within-group comparisons, only true acupuncture group showed a noticeable difference in depression after intervention (*P* = 0.022). For another one [27], effects were similar for both depression and anxiety in within-group comparisons, and also, there were no statistically significant differences between the true acupuncture group and the sham acupuncture group.

#### 3.4.3. Subgroup Analysis on Electroacupuncture

Descriptive analysis was adopted as data synthesis could not be conducted for subgroup analysis on electroacupuncture due to the insufficient number of studies and the inconsistent types of outcome data. Three studies [29, 32, 35] compared electroacupuncture with standard methods of treatment/care and the results were different. One trial [29] with a three-arm design indicated that both true electroacupuncture (*P* = 0.015) and sham electroacupuncture (*P* = 0.0088) presented a more effective improvement in HADS-D scores than wait-list control, while, for anxiety, only true intervention was found to be effective (*P* = 0.044). Meanwhile, this study [29] also found no significant improvement in sleep quality (as measured by PSQI) in both true and sham intervention groups, with the *P* values 0.058 and 0.31, respectively. However, based on the reported data, we still cannot judge whether true electroacupuncture is superior to the sham approach as between-groups comparisons were not reported in this article [29].

In another study [35], apart from the standard treatment/care in both groups, the intervention group adopted electroacupuncture plus music therapy to compare with the control arm using music therapy only, and the within-group analysis showed that the HAMD-D scores significantly decreased in both groups after the intervention with the *P* values, 0.013 and 0.022, respectively. However, between-groups comparisons revealed no statistical differences after the intervention (*P* = 0.431). In the study conducted by Frisk et al. [32], subscales of Women's Health Questionnaire (WHQ) and PGWB were employed to evaluate the status of depression and anxiety, and the subscale scores for anxiety (both WHQ and PGWB) were found to be improved significantly in the electroacupuncture group after the treatment, and all sleep parameters (including number of hours slept/night, times of wake-up/night, and WHQ sleep scores) also improved significantly in the electroacupuncture group (within-group comparison).

#### 3.4.4. Subgroup Analysis on Acupressure

There was only one trial [30] adopting acupressure as the intervention approach, and it was found that the acupressure group had lower anxiety and depression scores than the sham acupressure group, but these differences did not reach a significant level.

#### 3.4.5. Subgroup Analysis on Sham Acupoints Stimulation versus Wait-List Control 

Comparison between sham acupoints stimulation and wait-list control was only mentioned in one study [29]. In this study, the true electroacupuncture group selected the acupoints located around the joint with the most pain and the acupoints were inserted with needles until receiving “*de qi*,” while the sham comparison used nonpenetrating needles at nonacupuncture and nontrigger points located at least 5 cm from the joint without evoking the sensation of “*de qi*.” This study indicated that sham electroacupuncture can produce some treatment effects to improve the HADS-D scores in cancer patients (*P* = 0.0088), while, for anxiety and sleep quality, there were no significant differences between groups.

### 3.5. Adverse Events Associated with Acupoints Stimulation

Six trials [26, 27, 31–34] mentioned the potential adverse events as the safety outcomes, of which three studies [26, 31, 32] reported that there were no adverse events associated with acupoints stimulation. In Deng et al.'s study [27], a total of 11 serious side effects occurred during the intervention such as low blood counts, renal failure, and nausea, but all these negative events were deemed to be not related to acupuncture. The other two studies conducted by Molassiotis et al. [33, 34] monitored adverse events, but the one published in 2012 [33] did not report the details of adverse events in the study results, while the latter one, which was published in 2013 [34], reported that a small number of cases suffered from mild side effects including spot bleeding and minor pain/discomfort. None of the included trials assessed the causality between the intervention and the possible adverse events.

## 4. Discussion

In this systematic review, 11 trials with a total of 1073 participants were included. The study findings supported that acupoints stimulation can be a beneficial approach for managing anxiety and depression in cancer patients. However, some methodological flaws were still found in some studies. Considering the potential risks of bias identified in the analyzed trials and the limited number of included trials. The findings of this review should be interpreted prudently, and the current evidence on acupoints stimulation for anxiety and depression in cancer survivors can be rated as suggestive but still not fully conclusive.

Based on the overall analysis, acupoints stimulation as a complementary therapy could improve the mood of depression in patients with malignancy. The possible explanation for it might be that different levels of cortisol, epinephrine, and adrenocorticotropic hormone are associated with the negative moods in cancer patients, which are regulated by HPA and SNS, and acupoints stimulation could positively influence the HPA and SNS based on its potential immune-modulatory effect [24, 25]. It is noted that significant heterogeneity was identified in the overall assessment, and the possible explanations are as follows. Firstly, various types of acupoints stimulation were used among trials which more or less contribute to the heterogeneity in nature. Secondly, the measures for depression in the analyzed five studies were quite inconsistent, with three trials employing HAMD, and the other two applying HADS-D and BDI-PC, respectively, and the inconsistent outcome assessment tools could be another source for introducing the heterogeneity.

In the subgroup analyses, both manual acupuncture and electroacupuncture showed positive effects on depression when comparing with standard methods of treatment/care, among which two trials compared acupuncture with antidepressant, and one study [28] even demonstrated a more significant effect of acupuncture in relieving depression than using antidepressant. However, conclusion on the superiority of acupuncture to antidepressant should be interpreted with caution because some methodological flaws were identified in the mentioned trial such as the absence of blinding and allocation concealment and unbalanced cointerventions, all of which could increase the risks of bias inevitably.

For anxiety, encouraging results on acupoints stimulation were also reported in corresponding studies. Based on the results of data syntheses as well as descriptive analyses, acupoints stimulation can be a beneficial approach for relieving psychological distresses among oncology patients. However, the evidence concluded from the included studies is not fully conclusive at the current stage as a number of methodological flaws, such as the unclear statement of randomization, blinding and allocation concealment, and unbalanced cointerventions across groups, were identified. It is noted that the studies' effect size would be more inclined to be overestimated when the methodological quality of those studies is unsatisfactory [39].

Our study findings did not reach a conclusion on the evidence of acupoints stimulation for improving sleep quality for the following reasons: conflicting results were reported in the analyzed studies, the number of studies reporting sleep quality was quite limited, and those trials reporting sleep quality only included it as a secondary outcome. Effects of acupoints stimulation on sleep quality in cancer patients should be explored in future studies.

It is noted that several kinds of assessment tools, including CESD, HAMD, SDS, BDI-PC, and HADS, were used in the included studies for measuring anxiety and depression. These tools are generic instruments which can be applied in different populations, and their psychometric properties have also been well documented as valid and reliable measures used in cancer patients. For instance, Cronbach's alpha was greater than 0.75 for CESD [40], HAMD [41], SDS [42], and BDI-PC [43] which indicated a satisfactory internal consistency, and the construct validity of HADS was also tested through the factor analysis [38], and Cronbach's alphas for the anxiety and depression subscales were 0.887 and 0.703, respectively [44].

Placebo effects of acupoints stimulation should be explored in studies incorporating a true, sham, and usual care group. Based on the study findings, both true and sham acupoints stimulation can relieve anxiety and/or depression more effectively than standard methods of treatment/care, and the true interventions were somewhat better than the sham comparisons although the differences did not reach the statistical significance. Based on these findings, it seems that the treatment effects of acupoints stimulation may be associated with large placebo effects. According to a series of recent randomized controlled trials, placebo effects might partially contribute to the overall therapeutic effects of acupoints stimulation [45–48]. However, specific therapeutic effects of acupoints stimulation still play a crucial role in improving patients' functional status, as a large number of rigorously designed randomized controlled trials and systematic reviews have proved the superiority of true acupuncture/acupressure to the sham comparisons [49–52].

Several reasons could be employed to explain the nonsignificant differences between the true and sham acupoints stimulation in our study findings. Firstly, the nonspecific placebo effects might be exaggerated in the analyzed trials as the outcome measures for anxiety and/or depression were all scales measured by patients themselves or caregivers which can be easily affected by their expectancy to treatment, especially when the blinding design is not well performed. Secondly, it is still uncertain whether the sham procedures used in the included studies are really inert or not. One of the preconditions for developing a sham intervention is that it is indistinguishable from the true treatment but it should be inert in nature, which means having no specific therapeutic effects for the targeted health problem but only producing nonspecific physiological and/or psychological effects (placebo effects) [53]. In the analyzed trials, the design of sham acupoints stimulation might not be very appropriate. For example, nonspecific acupoints (one type of sham modality) which were close to the true acupoints might also produce some treatment effects, because, based on the holistic concept of Traditional Chinese Medicine (TCM) theory, acupoints stimulation performs its effects by regulating the whole body functions. The “nonacupoints” described in some trials might be potential, active acupoints which have not been identified on current acupoints chart. All of these designs could generate some specific treatment effects for patients in the sham intervention group. Moreover, in our included trials, only four incorporated a sham intervention arm. The relative small sample size and the methodological flaws identified in those trials made the final data analysis on sham intervention only a preliminary finding and not fully convincing.

Adverse events associated with acupoints stimulation were measured in six studies [26, 27, 31–34] and no serious harm data related to acupoints stimulation were reported. Acupoints stimulation could be a safe intervention used in clinical practice. Other systematic reviews, as well as prospective or retrospective surveys with a large sample size, also supported the idea that acupoints stimulation can be a relatively safe approach [54, 55]. However, it is noted that some analyzed studies in this review failed to include the safety issue as the outcome measure, and none of the included trials incorporated standardized criteria to assess the causality between the intervention and the reported adverse events.

Our findings suggested that acupoints stimulation can be a promising approach to managing the psychological distresses in cancer patients, but definite evidence still cannot be concluded from this review and the study finding can only be interpreted as suggestive due to the following limitations. Firstly, although the quality of the included studies was generally acceptable, methodological flaws still existed in some trials which could affect the reliability of our findings. Secondly, language bias cannot be excluded as all of the analyzed studies were only English or Chinese publications. In addition, publication bias might exist because there were seldom negative findings reported in our included studies, and we did not perform funnel plot as the number of trials included for each comparison was quite limited (less than ten).

## 5. Implications for Future Research and Practice

This study concluded several implications for future research and practice. Firstly, future research should elaborate more details on the protocols of acupoints stimulation in both true and sham intervention groups including the practitioner, duration of intervention and number of sessions, the selection and location of the true and sham acupoints, and the styles of stimulation. Secondly, apart from the certain psychological measures, some biomarkers [56] such as Interleukin-6 (IL-6), Interleukin-1*β* (IL-1*β*), and tumor necrosis factor-*α* (TNF-*α*) could be adopted as the potential indicators for emotional problems in future studies, which might minimize the exaggeration of treatment effects to some extent. Moreover, design of sham acupoints stimulation should be more reasonable; sham acupoints should be prudently selected to avoid choosing potentially effective true acupoints. An experienced TCM practitioner could be invited or consulted when developing the intervention protocol and some special-designed needles which cannot penetrate the acupoints could be adopted as the sham intervention. Furthermore, standard criteria should be applied to assess the causality between the adverse events and the acupoints stimulation. In addition, clinical researchers should take every effort to minimize the potential risks of bias. Although we understand it is difficult to reach blinding design among research personnel who conduct acupuncture or acupressure, some measures can still be considered to control the potential bias induced by study participants and outcome assessors in clinical settings; for example, researchers could use eye-patches to cover the participants' eyes when providing the intervention, and outcome assessors could be those people who are not involved in any other procedures of the study except for the data collection process [52, 57]. Finally, future studies should follow the CONSORT [58] and STRICTA [59] guidelines to enhance the report quality of the RCTs on acupoints stimulation.

## Figures and Tables

**Table 1:** Characteristics of included trials.

Study & setting	Types of cancer	Sample size	Acupoints stimulation intervention		Control	Primary and/or secondary outcomes^*∗*^
			Types of acupoints stimulation	Selected acupoints		
S^a^1: Bao et al., 2014 [26] Two cancer centers, USA	Breast cancer	*Randomized* = 51 *Completed* = 47 *Intervention group*: 23, age (yr) = median 61 (45–85) *Control group*: 24, age (yr) = Median 61 (44–82)	*Intervention*: manual acupuncture *Practitioner*: acupuncturist *Frequency*: weekly treatment *Total duration*: 8 weeks	Guanyuan (CV4), Qihai (CV6), Zhongwan (CV12), Hegu (LI4) Master of Heart 6 (MH6), Yanglingquan (GB34), Zusanli (ST36), Taixi (KI3), and Shugu (BL65)	*Sham acupuncture*: nonpenetrating retractable needles at 14 sham acupoints located at the midpoint of the line connecting 2 real acupoints	(i) *Depression*: Center for Epidemiologic Studies Depression Scale (CESD) (ii) *Anxiety*: Hospital Anxiety and Depression Scale (HADS-A) (iii) *Sleep quality*: Pittsburgh Sleep Quality Index (PSQI) (iv) *Adverse events*

S2: Deng et al., 2013 [27] A cancer center, USA	Various types of cancer	*Randomized* = 101 *Completed* = 74 *Intervention group*: 47, age (yr) = median 54 (IRQ 46, 58) *Control group*: 50, age (yr) = median 53 (IRQ 45, 59)	*Intervention*: manual acupuncture *Practitioner*: acupuncturist *Frequency*: weekly treatment *Total duration*: 6 weeks	Qihai (CV6), Guanyuan (CV4), Taixi (KI3), Zusanli (ST36), Sanyinjiao (SP6), Quchi (LI11), Yinxi (HT6), and auricular point for antidepression	*Sham acupuncture*: Blunt-tipped needles used at a few millimeters off the meridians and away from the points used in true acupuncture	(i) *Anxiety and Depression*: Hospital Anxiety and Depression Scale (HADS) (ii) *Adverse events*

S3: Feng et al., 2011 [28] Department of TCM, General Hospital of PLA, China	Various types of cancer	*Randomized* = 80 *Completed* = 80 *Intervention group*: 40, age (yr) = 63.80 ± 5.47 *Control group*: 40, age (yr) = 63.60 ± 4.26	*Intervention*: manual acupuncture *Practitioner*: acupuncturist *Frequency*: daily treatment *Total duration*: 30 days	Fenglong (ST40); Yinlingquan (SP9); Xuehai (SP10); Sanyinjiao (SP6); Yintang (EX-HN3); Baihui (DU20); Sishencong (EX-HN1); Neiguan (PC6); and Shenmen (TF4)	*Standard methods of treatment/care*: standard medication using antidepressant	(i) *Depression*: Self-Rating Depression Scale (SDS) and Hamilton Depression Scale (HAMD) (ii) *Sleep quality*: Pittsburgh Sleep Quality Index (PSQI)

S4: Mao et al., 2014 [29] A cancer center, USA	Breast cancer	*Randomized* = 67 *Completed* = 59 *Intervention group*: 22, age (yr) = 57.5 ± 10.1 *Control group 1*: 22 age (yr) = 60.9 ± 6.5 *Control group 2*: 23 age (yr) = 60.6 ± 8.2	*Intervention*: electroacupuncture *Practitioner*: acupuncturist *Frequency*: twice per week for 2 weeks and weekly treatment for the following 6 weeks *Total duration*: 8 weeks	At least 4 local points around the joint with the most pain and at least 4 distant points to address nonpain symptoms commonly observed in conjunction with pain	*Sham electroacupuncture (control 1)*: nonpenetrating needles at nonacupuncture, nontrigger points at least 5 cm from the joint where pain was perceived to be maximal *Wait-list control (control 2) *	(i) *Anxiety and depression*: Hospital Anxiety and Depression Scale (HADS) (ii) *Sleep quality*: Pittsburgh Sleep Quality Index (PSQI)

S5: Tang et al., 2014 [30] A medical center, Taiwan	Lung cancer	*Randomized* = 57 *Completed* = 45 *Group 1*: 17, age (yr) = 53.9 ± 9.8 *Group 2*: 24, age (yr) = 54.8 ± 9.5 *Group 3*: 16, age (yr) = 66.1 ± 8.0	*Intervention*: acupressure *Practitioner*: research assistant with acupressure training *Frequency*: daily treatment *Total duration*: 5 months	Hegu (LI4); Zusanli (ST36); Sanyinjiao (SP6)	*Sham acupressure*: sham acupressure at sham acupoints located at first metacarpal head, patella, and inner ankle	(i) *Anxiety and depression*: Hospital Anxiety and Depression Scale (HADS) (ii) *Sleep Quality*: Pittsburgh Sleep Quality Index (PSQI)

S6: Walker et al., 2010 [31] Oncology clinics, USA	Breast cancer	*Randomized* = 50 *Completed* = 27, age (yr) = median 55 (35–77) *Intervention group*: 25 *Control group*: 25	*Intervention*: manual acupuncture *Practitioner*: not reported *Frequency*: twice per week for the first 4 weeks and once per week for the following 8 weeks *Total duration*: 12 weeks for intervention and 1 year for follow-up	Kidney 3; Urinary bladder 23; Spleen 6; Gallbladder 20; Du 14; Du 20; Stomach 36; Liver 3; Heart 7; Pericardium 7; Ren 6; Lung 9	*Standard methods of treatment/care*: standard medication using antidepressant	(i) *Depression*: Beck Depression Inventory-Primary Care (BDI-PC) (ii) *Adverse events*

S7: Frisk et al., 2012 [32] Three participating centers, Sweden	Breast cancer	*Randomized* = 45 *Completed* = 18 *Intervention group*: 26 age (yr) = mean 54.1 (47–69) *Control group*: 18 age (yr) = mean 53.4 (43–67)	*Intervention*: electroacupuncture *Practitioner*: physiotherapist *Frequency*: twice a week the first two weeks and then once a week for 10 weeks *Total duration*: 12 weeks	Xinshu (BL15); Shenshu (BL23); Ciliao (BL32); Baihui (GV20); Shenmen (HE7); Neiguan (PC6); Taichong (LR3); Sanyinjiao (SP6); Yinlingquan (SP9)	*Standard methods of treatment/care*: standard medication (sequential or continuous combined estrogen/progestagen therapy for hot flushes)	(i) *Depression/anxiety*: Psychological and General Well-Being Index (PGWB) and Women's Health Questionnaire (WHQ) (ii) *Sleep data*: the numbers of times wake-up/night and hours of sleep (iii) *Adverse events*

S8: Molassiotis et al., 2012 [33] Two cancer hospitals, four cancer centers, and three treatment centers of a national voluntary breast cancer organization, United Kingdom	Breast cancer	*Randomized* = 302 *Completed* = 246 *Intervention group*: 227, age (yr) = mean 52 (30–75) *Control group*: 75, age (yr) = mean 53 (25–80)	*Intervention*: manual acupuncture *Practitioner*: therapists with acupuncture training *Frequency*: 6 sessions with each session lasting 20 min *Total duration*: 6 weeks	Zusanli (ST36); Sanyinjiao (SP6); Hegu (LI4); and some alternative points [Yanglingquan (GB34) and Yinlingquan (SP9)]	*Standard methods of treatment/care*: enhanced usual care with information booklet on how to cope with fatigue	(i) *Anxiety and depression*: Hospital Anxiety and Depression Scale (HADS) (ii) *Adverse events*

S9: Molassiotis et al., 2013 [34] Two cancer hospitals, four cancer centers, and three treatment centers of a national voluntary breast cancer organization, United Kingdom	Breast cancer	*Randomized* = 198 *Completed* = 151, age (yr) = mean: 53 *Intervention group*: 65 *Control group 1*: 67 *Control group 2*: 65	*Intervention*: manual acupuncture *Practitioner*: acupuncturist *Frequency*: weekly treatment *Total duration*: 4 weeks	Zusanli (ST36); Sanyinjiao (SP6); Hegu (LI4)	*Self-acupuncture (control 1)*: self-acupuncture at Zusanli (ST36) and Sanyinjiao (SP6) *No acupuncture (control 2)*	(i) *Anxiety and depression*: Hospital Anxiety and Depression Scale (HADS) (ii) *Adverse events*

S10: Xiang et al., 2006 [35] A TCM hospital, Beijing, China	Various types of cancer (details not given)	*Randomized* = 92 *Completed* = 92 *Intervention group*: 46, age (yr) = 41–82 *Control group*: 46, age (yr) = 37–89	*Intervention*: electroacupuncture + music therapy *Practitioner*: doctor *Frequency*: daily treatment *Total duration*: 4 weeks	Neiguan (PC6); Zusanli (ST36); Sanyinjiao (SP6); Baihui (DU20); Taixi (KI3); Yintang (EX-HN3); and so forth.	*Music therapy + standard methods of treatment/care* (usual care)	*Depression*: Hamilton Depression Scale *(HAMD)* and Self-Rating Depression Scale *(SDS)*

S11: Shi et al., 2013 [36] A hospital, Anhui, China	Gynecological cancer	*Randomized* = 30 *Completed* = 30 *Intervention group*: 15, age (yr) = 43.3 ± 1.94 *Control group*: 15, age (yr) = 45.0 ± 1.49	*Intervention*: manual acupuncture *Practitioner*: not reported *Frequency*: twice per week *Total duration*: 3 months	Neiguan (PC6); Zusanli (ST36); Sanyinjiao (SP6); Taichong (LR3); Hegu (LI4); Guanyuan (RN4); Qihai (RN6); Taixi (KI3); Shenshu (BL23); Ganshu (BL18); Pishu (BL20)	*Standard methods of treatment/care*: usual care	*Depression*: Hamilton Depression Scale (HAMD)

^a^S = study, ^*∗*^Primary and/or secondary outcomes which were determined by the systematic review.

**Table 2:** Methodological and quality assessment of included trials.

Criteria	S^a^1	S2	S3	S4	S5	S6	S7	S8	S9	S10	S11
(1) “Was the method of randomization adequate?”	✓	✓	✓	✓	✓	?	✓	✓	✓	✓	?
(2) “Was the treatment allocation concealed?”	*✗*	✓	?	✓	?	?	✓	✓	✓	?	?
(3) “Was the patient blinded to the intervention?”	✓	✓	?	✓	✓	?	?	*✗*	*✗*	?	?
(4) “Was the care provider blinded to the intervention?”	*✗*	*✗*	?	*✗*	*✗*	?	?	*✗*	*✗*	?	?
(5) “Was the outcome assessor blinded to the intervention?”	✓	✓	?	✓	✓	?	?	*✗*	*✗*	?	?
(6) “Was the drop-out rate described and acceptable?”	✓	✓	✓	✓	✓	✓	*✗*	✓	✓	✓	✓
(7) “Were all randomized participants analyzed in the group to which they were allocated?”	✓	✓	✓	✓	✓	✓	*✗*	*✗*	*✗*	✓	✓
(8) “Are reports of the study free of suggestion of selective outcome reporting?”	✓	✓	✓	✓	✓	?	✓	✓	✓	✓	✓
(9) “Were the groups similar at baseline regarding the most important prognostic indicators?”	✓	✓	✓	✓	✓	✓	✓	✓	✓	✓	✓
(10) “Were co-interventions avoided or similar?”	✓	✓	?	✓	?	?	✓	?	?	✓	?
(11) Was the compliance acceptable in all groups?	?	?	?	✓	✓	?	✓	✓	✓	?	?
(12) “Was the timing of the outcome assessment similar in all groups?”	✓	✓	✓	✓	✓	✓	✓	✓	✓	✓	✓
(13) “Are other sources of potential bias unlikely?”	?	?	?	?	?	?	?	?	?	?	?

Based on [37].

^a^S: study, ✓: low risk of bias; *✗*: high risk of bias; ?: unclear risk of bias.

**Table 3:** Summary of meta-analyses.

Study outcomes	Number of trials	Number of participants	Statistical method	Effect estimate	Test for overall effect		Heterogeneity (*I* ^2^)
					*Z*	*P*	
Overall assessment of acupoints stimulation: acupoints stimulation versus different comparisons (for anxiety)							
Acupoints stimulation versus standard methods of treatment/care	3	567	NR	NR	NR	NR	NA
Acupoints stimulation versus sham acupoints stimulation	2	137	Mean difference (IV, random, 95% CI)	−0.65 [−2.84, 1.53]	0.59	0.56	>50%

Overall assessment of acupoints stimulation: acupoints stimulation versus different comparisons (for depression)							
Acupoints stimulation versus standard methods of treatment/care	5	485	Mean difference (IV, random, 95% CI)	−1.08 [−1.97, −0.19]	2.39	0.02	>50%
Acupoints stimulation versus sham acupoints stimulation	2	137	Mean difference (IV, random, 95% CI)	−0.27 [−2.66, 2.12]	0.23	0.82	50%

Subgroup analysis: different types of acupoints stimulation versus standard methods of treatment/care (for anxiety)							
Electroacupuncture	2	112	NR	NR	NR	NR	NA
Acupuncture	2	302^*∗*^	NR	NR	NR	NR	NA

Subgroup analysis: different types of acupoints stimulation versus standard methods of treatment/care (for depression)							
Electroacupuncture	2	159	NR	NR	NR	NR	NA
Acupuncture	4	393	Mean difference (IV, random, 95% CI)	−2.55 [−3.61, −1.49]	4.72	<0.00001	>50%

Subgroup analysis: different types of acupoints stimulation versus sham comparison (for anxiety)							
Acupuncture	2	152	NR	NR	NR	NR	NA
Acupressure	1	57	NR	NR	NR	NR	NA

Subgroup analysis: different types of acupoints stimulation versus sham comparison (for depression)							
Acupuncture	2	152	NR	NR	NR	NR	NA
Acupressure	1	57	NR	NR	NR	NR	NA

IV: inverse variance, CI: confidence interval, NR: not reported because pooling was not conducted due to the insufficient number of studies, different models of data, or absence of data, NA: not applicable

^*∗*^Sample was adopted from one study (as the sample of the other study was based on the study we adopted).

**Table 4:** Selected search strategies.

ID	Searching strategies	Records
PubMed		
#1	Search “acupuncture”[MeSH Terms]	17708
#2	Search “acupuncture therapy”[MeSH Terms]	16944
#3	Search “acupressure”[MeSH Terms]	465
#4	Search “acupuncture, ear”[MeSH Terms]	267
#5	Search “acupuncture points”[MeSH Terms]	4063
#6	Search “electroacupuncture”[MeSH Terms]	2566
#7	Search “auriculotherapy”[MeSH Terms]	277
#8	#1 OR #2 OR #3 OR #4 OR #5 OR #6 OR #7	17958
#9	Search ((((((((acupunctur^*∗*^[Title/Abstract]) OR acupressure^*∗*^[Title/Abstract]) OR auriculotherap^*∗*^[Title/Abstract]) OR electroacupunctur^*∗*^[Title/Abstract]) OR acupoint^*∗*^[Title/Abstract]) OR needl^*∗*^[Title/Abstract]) OR acupuncture therapy[Title/Abstract]) OR “ear acupuncture”[Title/Abstract]) OR acupuncture points[Title/Abstract]	104918
#10	#8 or #9	108214
#11	Search “neoplasms”[MeSH Terms]	2609885
#12	Search ((((((((cance^*∗*^[Title/Abstract]) OR carcinom^*∗*^[Title/Abstract]) OR neoplasm^*∗*^[Title/Abstract]) OR tumo^*∗*^[Title/Abstract]) OR tumou^*∗*^[Title/Abstract]) OR neoplasia[Title/Abstract]) OR oncolog^*∗*^[Title/Abstract]) OR malignan^*∗*^[Title/Abstract]) OR neoplasms[Title/Abstract]	2345516
#13	#11 OR #12	3231222
#14	Search “anxiety”[MeSH Terms]	56178
#15	Search “anxiety disorders”[MeSH Terms]	67844
#16	Search “depression”[MeSH Terms]	153512
#17	Search “depressive disorder”[MeSH Terms]	80897
#18	Search “mood disorders”[MeSH Terms]	115156
#19	Search “affective disorders, psychotic”[MeSH Terms]	33045
#20	Search “stress, psychological”[MeSH Terms]	92744
#21	#14 OR #15 OR #16 OR #17 OR #18 OR #19 OR #20	346299
#22	Search (((((((anxiet^*∗*^[Title/Abstract]) OR anxiou^*∗*^nervousness[Title/Abstract]) OR hypervigilanc^*∗*^[Title/Abstract]) OR depression^*∗*^[Title/Abstract]) OR depressiv^*∗*^[Title/Abstract]) OR emotion^*∗*^[Title/Abstract]) OR psycholog^*∗*^[Title/Abstract]) OR affective^*∗*^[Title/Abstract]	518732
#23	#21 OR #22	687179
#24	#10 AND #13 AND #23	251

Cochrane Central Register of Controlled Trials (CENTRAL)		
#1	MeSH descriptor: [Acupuncture Therapy] explode all trees	3132
#2	MeSH descriptor: [Acupuncture] explode all trees	151
#3	MeSH descriptor: [Acupressure] explode all trees	227
#4	MeSH descriptor: [Acupuncture Points] explode all trees	1069
#5	MeSH descriptor: [Acupuncture, Ear] explode all trees	123
#6	MeSH descriptor: [Auriculotherapy] explode all trees	129
#7	MeSH descriptor: [Electroacupuncture] explode all trees	473
#8	acupunctur^*∗*^ or acupressure^*∗*^ or auriculotherap^*∗*^ or electroacupunctur^*∗*^ or acupoint^*∗*^ or needl^*∗*^:ti,ab,kw (Word variations have been searched)	13648
#9	#1 or #2 or #3 or #4 or #5 or #6 or #7 or #8	13710
#10	MeSH descriptor: [Neoplasms] explode all trees	52791
#11	neoplasm^*∗*^ or tumo^*∗*^ or tumou^*∗*^ or neoplasia or cance^*∗*^ or carcinom^*∗*^ or malignan^*∗*^ or oncolog^*∗*^:ti,ab,kw (Word variations have been searched)	94737
#12	#10 or #11	100138
#13	MeSH descriptor: [Anxiety] explode all trees	5187
#14	MeSH descriptor: [Anxiety Disorders] explode all trees	4954
#15	MeSH descriptor: [Depression] explode all trees	5472
#16	MeSH descriptor: [Affective Disorders, Psychotic] explode all trees	1648
#17	MeSH descriptor: [Emotions] explode all trees	11649
#18	anxiet^*∗*^ or anxiou^*∗*^ or nervousness or hypervigilanc^*∗*^ or depression^*∗*^ or depressiv^*∗*^ or emotion^*∗*^ or Psycholog^*∗*^ or affective^*∗*^:ti,ab,kw (Word variations have been searched)	102362
#19	#13 or #14 or #15 or #16 or #17 or #18	103970
#20	#9 and #12 and #19	108
#21	#20 in Tails	84
